# Identification and characterization of a virus-specific continuous B-cell epitope on the PrM/M protein of Japanese Encephalitis Virus: potential application in the detection of antibodies to distinguish Japanese Encephalitis Virus infection from West Nile Virus and Dengue Virus infections

**DOI:** 10.1186/1743-422X-7-249

**Published:** 2010-09-22

**Authors:** Rong-Hong Hua, Na-Sha Chen, Cheng-Feng Qin, Yong-Qiang Deng, Jin-Ying Ge, Xi-Jun Wang, Zu-Jian Qiao, Wei-Ye Chen, Zhi-Yuan Wen, Wen-Xin Liu, Sen Hu, Zhi-Gao Bu

**Affiliations:** 1 State Key Laboratory of Veterinary Biotechnology, Harbin Veterinary Research Institute, Chinese Academy of Agricultural Sciences, Harbin 150001, PR China; 2 State Key Laboratory of Pathogen and Biosecurity, Beijing Institute of Microbiology and Epidemiology, Beijing 100071, China

## Abstract

**Background:**

Differential diagnose of Japanese encephalitis virus (JEV) infection from other flavivirus especially West Nile virus (WNV) and Dengue virus (DV) infection was greatly hindered for the serological cross-reactive. Virus specific epitopes could benefit for developing JEV specific antibodies detection methods. To identify the JEV specific epitopes, we fully mapped and characterized the continuous B-cell epitope of the PrM/M protein of JEV.

**Results:**

To map the epitopes on the PrM/M protein, we designed a set of 20 partially overlapping fragments spanning the whole PrM, fused them with GST, and expressed them in an expression vector. Linear epitope M14 (^105^VNKKEAWLDSTKATRY^120^) was detected by enzyme-linked immunosorbent assay (ELISA). By removing amino acid residues individually from the carboxy and amino terminal of peptide M14, we confirmed that the minimal unit of the linear epitope of PrM/M was M14-13 (^108^KEAWLDSTKAT^118^). This epitope was highly conserved across different JEV strains. Moreover, this epitope did not cross-react with WNV-positive and DENV-positive sera.

**Conclusion:**

Epitope M14-13 was a JEV specific lineal B-cell epitpe. The results may provide a useful basis for the development of epitope-based virus specific diagnostic clinical techniques.

## Background

Japanese encephalitis virus (JEV) is the most important cause of epidemic encephalitis in most Asian regions. The virus belongs to the genus *Flavivirus *of the family *Flaviviridae*; about 35,000-50,000 cases of and 10,000 deaths from JEV infection are reported annually [1]. JEV was first isolated in Japan in 1935, following which it spread to most other Asian countries. At present, this virus is even found in regions beyond its ecological boundaries. Recently, JEV has spread to regions as far as northern Australia [2,3]. Hence, there is a concern that JEV might become a global threat. In fact, it is not unusual to find 2 or more flaviviruses co-circulating in one area. In Southeast Asia, the most important flaviviruses are JEV and dengue viruses (DENV) [4]. In northern Australia, Kunjin virus is found to co-circulate with JEV [5]. In Vladivostok, Russia, studies have reported the detection of WNV in birds [6]. In addition, there is evidence that WNV infection in India from Japanese encephalitis nonendemic areas and endemic areas [7].

The flaviviruses WNV, DENV, and JEV share some common features, such as transmission via mosquitoes, and cross-react with each other in serological tests. These cross-reactive responses could confound the interpretation during serological testing, including neutralization tests and enzyme-linked immunosorbent assay (ELISA) [8]. JEV contains a single-stranded, positive-sense RNA genome with a size of 11 kb; the genome encodes 3 structural proteins, namely, core protein (C), premembrane protein (prM/M), and envelope protein (E), and 7 nonstructural proteins (NS1, NS2A, NS2B, NS3, NS4A, NS4B, NS5). Of the 10 proteins, the E protein is the dominant antigen responsible for eliciting neutralizing antibodies and plays an important role in inducing immunologic responses in the infected host. However, antibodies against the E proteins of the 3 aforementioned flaviviruses could cross-reactive with each other. Previous reports [9,10] show that in western blot (WB) prM protein may be used to serologically differentiate individuals infected with JEV from those infected with DENV, WNV and SLEV. Our preliminary WB results for JEV-positive sera also showed that prM reactivity could be used to differentiate JEV-positive sera from WNV- and DENV-positive sera. Hence, prM and antibodies against prM would be useful for conducting seroepidemiological studies of flavivirus infections in the regions that have prevalence of more than one flavivirus. However, because prM is a membrane protein, it is difficult to express it in *Escherichia coli *or other expression systems. In this report, we mapped and identified a linear B-cell epitope on the prM/M protein of JEV.

## Results

### Mapping of antigenic epitopes on PrM/M protein of JEV

**Figure 1:**
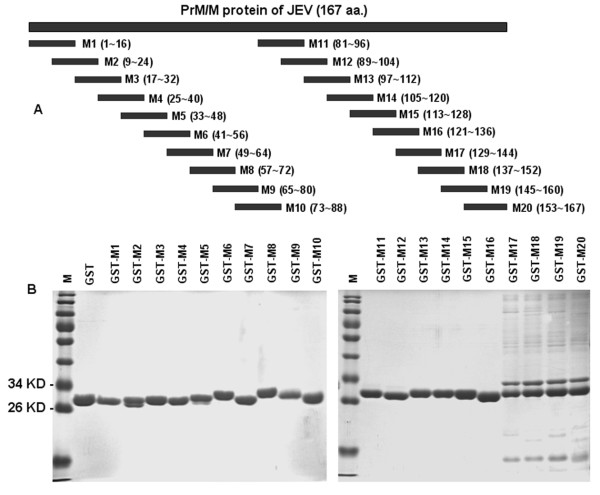
**Short peptide designing, expression and purification**. (A) Schematic diagram of the relative location of the truncated prM/M protein fragments and overlapping short peptides, M1-M20, spanning the prM/M protein. The numbers in parentheses indicate the amino acids located at the beginning or the end of each fragment. M1 to M20 are a set of partially overlapping short peptides covering the whole prM/M protein of JEV. (B) Expression and purification of recombinant peptide fusion proteins. For each peptide a fusion expression recombinant plasmid was constructed and transformed into host cell *E. coli *BL21 (DE3). After the cells were induced with IPTG, the supernatants of the sonicates were purified by affinity chromatography. The purified proteins were analyzed by 12% SDS-PAGE and stained with Coomassie brilliant blue. M stands for the protein molecular standards as labeled on the left.

**Figure 2:**
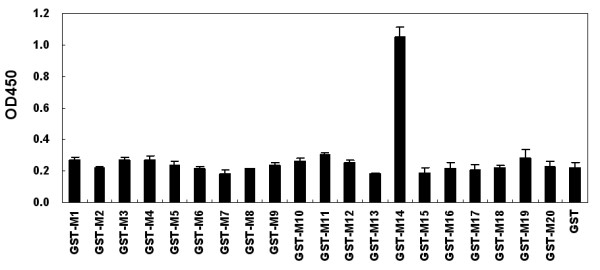
**Identification of the antigenic determinants on the prM/M protein with JEV-positive swine sera**. (A) ELISA analysis of the short fusion peptides. Microtiter plates were coated with purified recombinant fusion protein samples (2 μg/100 μl per well). The plate was blocked with skim milk and pooled JEV-positive swine sera (dilution, 1:200) was added, following which HRP-coupled goat anti-pig IgG secondary antibody was added. Only peptide M14 showed strong reactivity with the JEV-positive swine sera. (B) Western blot analysis confirmed the result of ELISA. JEV-positive swine sera could react with GST-M14 but not with other peptide fusion protein and GST.

To map the antigenic epitope of the JEV PrM/M protein, 20 partially overlapping 16-amino-acid long fragments (M1-M20) were designed (M20 was 15-amino-acid long) spanning the entire length of the PrM/M protein (Fig. 1A). All the fragments were fused with GST and expressed in the pGEX-6p-1 vector. The recombinant fusion proteins were purified with Glutathione Sepharose 4B RediPack column affinity chromatography according to the manufacturer's instructions (Amersham-Pharmacia Biotech) (Fig. 1B). Indirect ELISA and western blot assays with pooled JEV-positive swine sera were performed for antigenicity analysis of the 20 recombinant fusion proteins. Both ELISA (Fig. 2) and western blot (data not shown) results revealed that the peptide M14 was recognized by the JEV-positive swine sera.

### Epitope M14 was JEV specific

**Figure 3:**
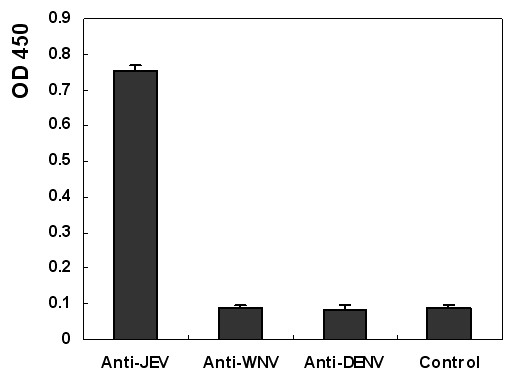
**The epitope did not react with WNV-positive and DENV-positive sera**. The synthesized epitope peptide was used as coating antigen in indirect ELISA. The reactivity of the epitope with the rabbit sera against JEV and that of the rabbit sera against DENV were assessed. Rabbit sera against JEV and normal rabbit sera were used as the positive and negative controls, respectively.

To investigate whether the anti-DENV or anti-WNV sera could recognize the M14 epitope, we synthesized an epitope peptide VNKKEAWLDSTKATRY and used it as coating antigen for indirect ELISA with anti-DENV and anti-WNV sera. The result showed that epitope M14 could only be recognized by anti-JEV sera but not by anti-DENV and anti-WNV sera (Fig. 3).

**Figure 4:**
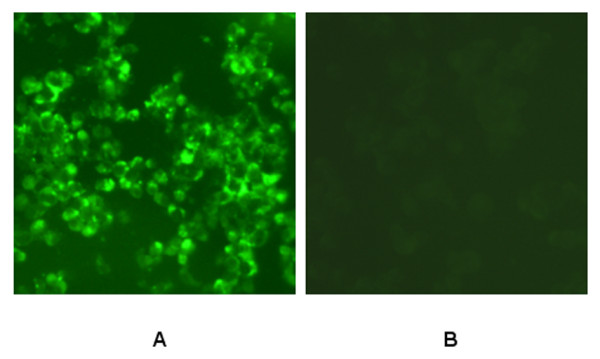
**Immunofluorescence analysis of epitope-specific mice monoclonal antibody 2F7**. Immunofluorescence assay of the harvested and fixed BHK21 cells infected with JEV with epitope-specific MAb 2F7 as the primary antibody and a FITC-conjugated goat anti-mouse IgG as the secondary antibody revealed that MAb recognized JEV infected BHK21 cells (A) but not uninfected cells (B).

The epitope M14-specific MAb 2F7 was generated by using epitope fusion protein GST-M14 as the antigen for immunofluroscence assay and synthesized epitope M14 peptide as the antigen for indirect ELISA. To investigate whether the MAb 2F7 could recognize the native PrM/M protein in infected cells, we stained the infected BHK-21 cells with the MAb 2F7. We found that MAb 2F7 could stain JEV-virus infected cells but not uninfected cells (Fig. 4A, 4B). Moreover, in the western blot analysis, the MAb 2F7 antibody recognized a 19-kd PrM/M band (data not shown). These results demonstrated that the linear continuous epitope M14 was expressed on the surface of JEV. Both Western blot and immunofluorescence assays revealed that the epitope-specific MAb recognized the native PrM/M protein.

### Fine mapping of the epitope

**Figure 5:**
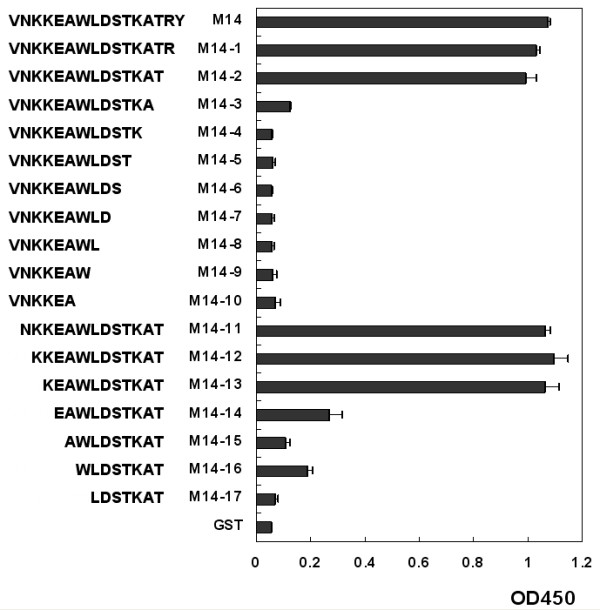
**Finer mapping the epitope M14-13**. Peptide M14 was truncated from the carboxy and amino terminal. After short protein fragments were fusion expressed with GST, they were analyzed by ELIZA using MAb 2F7. When 2 or more amino acid residues were removed from the carboxy terminal and 4 or more amino acid residues were removed form the amino terminal, the reactivity between MAb 2F7 and peptide fusion protein decreased greatly. This result shows that peptide M14-13 (KEAWLDSTKAT) is the minimal requirement for MAb 2F7 to recognize the linearized epitope.

To precisely locate the core sequence of epitope M14 on the PrM/M protein, a series of truncated peptides were designed and expressed after fusion with GST. First, the amino acid residues were removed one by one from the carboxy terminal of peptide M14 until the peptide was only 6-amino-acid long. The shortened peptide fusion protein was analyzed by ELISA using MAb 2F7. The result showed that when 3 or more amino acid residues were removed, the reactivity between MAb 2F7 and peptide fusion protein decreased completely (Fig. 5). Next, the amino acid residues were removed one by one from the amino terminal of peptide M14-2 until the peptide was only 6-amino-acid long. The shortened peptide fusion proteins were also analyzed by ELISA using MAb 2F7. We found that when 4 or more amino acid residues were removed from the amino terminal, the reactivity between MAb 2F7 and the peptide fusion protein decreased greatly. Taken together, these results demonstrated that peptide M14-13 (KEAWLDSTKAT) is the minimal requirement for MAb 2F7 to recognize the linearized epitope.

### Homology and cross-reactivity analysis

**Figure 6:**
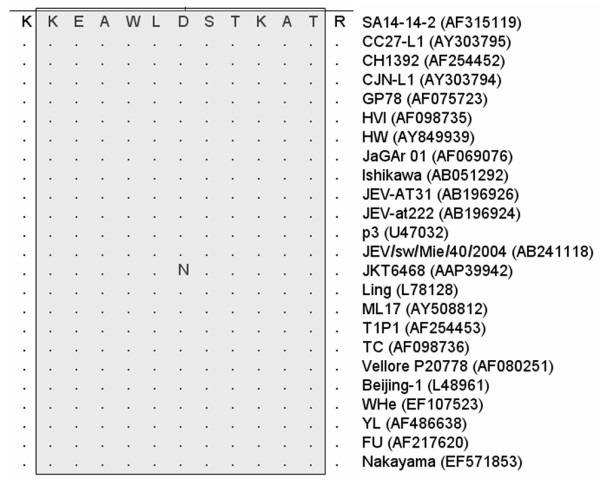
**Sequence alignment of the epitope M14-13 with different JEV strains**. Full-length sequences of 23 different JEV strains were selected from the GenBank. GenBank accession numbers are listed in the parentheses. Epitope M14-13 was highly conserved across these JEV strains.

**Figure 7:**
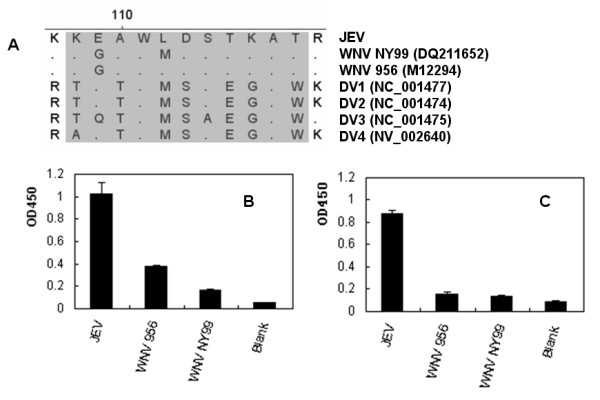
**Specificity analysis of the epitope**. (A) Sequence alignment between the identified linear epitope and the homologous regions of WNV and DENV. The virus strains listed in this figure were selected as representative strains. Abbreviations of each virus and GenBank accession numbers are listed in the parentheses. (B, C) The epitope homology peptides of WNV were expressed and analyzed by ELISA using MAb 2F7. The homology peptide of WNV strain NY99 did not react with MAb 2F7, while the homology peptide of WNV strain 956 was weakly reactive with MAb 2F7 (B). JEV-positive swine sera also did not reactive with the homology peptide of WNV (C).

To analyze the homology among the linear PrM/M protein epitopes, we used the PrM/M protein sequences from 24 different JEV strains for alignment analysis. Of the 24 JEV strains, all genotype 4 JEV were included. The alignment analysis revealed that the sequence of the linear epitope is greatly conserved across the JEV strains, except in the JKT6468 strain that had a mutation (D to N) (Fig. 6). In fact, the further survey showed that the substitution at this amino acid residue did not affect the binding ability of MAb 2F7 to the epitope. Sequence alignment was also performed between the epitope and the homologous sequences of other 2 flaviviruses, i.e., WNV and DENV (Fig. 7A). The homology between epitope M14-13 and those from the 4 serotype DENV was less than 40%. The homology between epitope M14-13 and WNV (including 2 lineages) was more than 80%. After the homologous sequences of WNV were fusion expressed with GST, none of the sequences showed reactivity with JEV-positive swine sera (Fig. 7C) by ELISA. And for MAb only peptide lineage II strain 956 WNV showed weakly reactive with MAb 2F7 and epetide lineage I strain NY99 did not reactive with MAb 2F7 (Fig. 7B).

## Discussion

In this study, a series of overlapping 16-amino-acid long peptides spanning the whole PrM/M protein of JEV were fused expressed with GST. Using the GST fusion protein set and the pooled JEV-positive swine sera, we mapped the linear epitope of the JEV PrM/M protein. The ELISA results indicated that the epitope was located in the M14 region (^105^VNKKEAWLDSTKATRY^120^). Moreover, by individually removing amino acid residues from the carboxy and amino terminals of the peptide M14, we confirmed that the minimal unit of the linear epitope of PrM/M was M14-13 (^108^KEAWLDSTKAT^118^). Sequence alignment showed that the epitope was greatly conserved across different JEV strains. Of the 24 selected JEV strains, only 1, i.e., strain JKT6468--a genotype 4 JEV strain--has one amino acid residue (^113^N) different from that of the epitope M14-13 (^113^D). However, further analysis revealed that this amino acid residue replacement (^113^D to ^113^N) did not affect the reactivity of the epitope to MAb 2F7- and JEV-positive swine sera.

Cross-reactivity among flaviviruses has been a great diagnostic challenge, especially for members of the JE serocomplex, in which it is difficult to differentiate between the strains by using ELISA or even by neutralization tests or other serological methods [11,12]. Serological cross-reactivity was mainly observed because these virus antigens contain the same immunodominant E glycoprotein epitopes responsible for eliciting a large proportion of cross-reactive serum antibodies during viral infection [13]. In this study, we identified a linear epitope from the PrM/M protein; this epitope could react with the JEV-positive swine sera and rabbit sera but not with rabbit anti-DENV and anti-WNV sera. The homogeneity between this linear epitope and all 4 serotype DENV PrM/M protein was less than 40%. Moreover, the same domains of the PrM/M protein from WNV did not react with the swine-anti JEV sera. and MAb 2F7. And only peptide lineage II strain 956 WNV showed weakly reactive with MAb 2F7 and epetide lineage I strain NY99 did not reactive with MAb 2F7. However around the world the predominant WNV were lineage I and lineage II consists exclusively of viruses from southern Africa and Madagascar and rare Europe district [14]. Hence, we confirmed that the epitope M14-13 was virus specific and could be potentially used in serological monitoring and for differential diagnosis of JEV infection.

Epitope identification was useful for elucidating the antigenic characteristics of the prM protein. The epitopes on E protein [15] and NS1 protein [16] of JEV have been mapped. However, thus far, the epitope on the PrM/M protein of JEV has not been mapped. Here, we first mapped the epitope on the PrM/M protein and identified a JEV-specific and highly conserved linear B-cell epitope precisely on the JEV PrM/M protein. It becomes difficult to purify and express membrane proteins in *E. coli*. However, the epitope on the JEV PrM/M protein can be easily fusion expressed with a vector protein or can be coupled with a vector protein after synthesis. This is the first report on the generation of MAbs against the PrM/M protein using epitope fusion as the immune antigen.

## Conclusions

The present study fully mapped the lineal eiptope on PrM/M protein of JEV and identified one lineal B-cell epitope M14-13. The epitope M14-13 was JEV specific and highly conservec among different JEV strains. These data could provide important basis for the potential application of epitope-based virus-specific diagnosis and would also be useful for further elucidating antigenic structure and biological function of PrM/M of JEV.

## Methods

### Cell lines, viruses, and serum specimens

Baby hamster kidney (BHK-21) cells and SP2/0 myeloma cells were cultured in RPMI-1640 medium supplemented with 10% fetal calf serum and antibiotics. All the cells were maintained in humidified 5% CO_2 _atmosphere at 37°C. The JEV strain SA14-14-2 [GenBank: AF315119] was propagated in BHK-21 cells to prepare the antigen for WB and immunofluorescence assays. The JEV-positive sera were obtained from pigs reared in pig farms. The sera were first tested by indirect ELISA and latex agglutination test (LAT) for detecting antibodies to JEV. Hyperimmune sera against JEV, WNV, and DENV were obtained from rabbits that had been inoculated with purified JEV, WNV, and DENV, respectively.

### Expression and purification of overlapping short fragments covering the PrM/M protein

To map the epitope on the PrM/M protein of JEV, we designed a set of 20 partially overlapping short peptides M1-M20 covering the whole PrM/M protein as shown in fig 1A. For each short peptide, a pair of oligonucleotide strands was synthesized. The 2 strands were annealed, and the resultant double-stranded DNA contained a *Bam*HI and an *Xho*I cohesive terminus at the 5' and 3' ends, respectively. The annealed fragment was cloned into the expression vector pGEX-6p-1. The inserts in the recombinant plasmids were sequenced, and the confirmed recombinant plasmids were transformed into *E. coli *strain BL21 and induced with 0.1 M isopropyl-D-thiogalactopyranoside (IPTG) at 37°C for 4 h. The expressed recombinant fusion proteins were analyzed with sodium dodecyl sulfate (SDS)-polyacrylamide gel electrophoresis (PAGE). Next, the fusion proteins were purified by Glutathione Sepharose 4B RediPack Column affinity chromatography according to the manufacturer's instructions (Amersham Pharmacia Biotech). Subsequently, the bound fusion protein was eluted with glutathione elution buffer (10 mM reduced glutathione, 50 mM Tris-HCl, pH 8.0) for further analysis.

### Enzyme-linked immunosorbent assay (ELISA)

Ninety-six-well microtiter plates were coated with purified fusion protein or denatured inclusion body in 0.1 M carbonate buffer (pH 9.6) at 4°C overnight and blocked with 5% skim milk for 3 h. Subsequently, the plates were washed 3 times with PBST (phosphate buffered saline, PBS with 0.1% Tween 20). In the binding assay, the plates were incubated with diluted rabbit hyperimmune sera or swine sera at 37°C for 1 h followed by washing 3 times with PBST. Bound antibodies were detected with horseradish peroxidase (HRP)-conjugated secondary antibody. The reaction was stopped with 2 M H_2_SO_4_, and the absorbance was measured at 450 nm by using a microplate autoreader (Bio-Rad). In peptide ELISA, microtiter plates were coated with the synthesized peptide.

### Western blotting

Virus-infected cell lysates or expressed fusion proteins were mixed with an equal volume of sample loading buffer (50 mM Tris-HCl, pH 6.8; 100 mM dithiothretol (DTT); 2% SDS; 0.1% bromophenol blue; and 10% glycerol) and separated by 12% SDS-PAGE. For immunoblotting, the proteins were transferred from the SDS-polyacrylamide gel to a nitrocellulose membrane. Non-specific antibody binding sites were blocked with 5% skim milk in PBS overnight at 4°C. The membranes were incubated with primary antibody at 37°C for 1 h and then washed 3 times with PBST (10 min each time). Next, the blot was probed with an appropriate secondary antibody (1:5,000) for 1 h at 37°C. The following secondary antibodies were used: horse radish peroxidase (HRP)-conjugated goat anti-mouse IgG (Sigma) and HRP-conjugated goat anti-pig IgG (Beijing Zhongshang biotechnology Co., Ltd.). After incubation with the secondary antibody, each blot was washed 3 times with PBST, and then developed with HRP developer.

### Generation of monoclonal antibody (MAb)

BALB/c mice were immunized with 250 μg epitope fusion protein GST-M14 in 0.5 ml emulsion with complete Freund's adjuvant. At 2 weeks interval, 2 booster injections with the same dose of emulsion were administered as the first immunization. At 3 days after the last injection, the spleens were surgically removed from the mice. Splenocytes were fused with SP2/0 myeloma cells as described previously [17]. Hybridoma clones were screened by indirect ELISA using synthesized epitope peptides M14 as the coating antigen. Selected clones were subcloned by limiting dilution. The resultant hybridoma clones were isotyped using an isotyping kit from Roche Diagnostics. Ascites fluids were produced in pristine-primed BALB/c mice.

### Immunofluorescence assays

BHK-21 cells were infected with JEV strain SA14-14-2 and incubated for 72 h at 37°C. The cells were harvested by scraping, centrifuged, and washed twice in PBS. Glass slides were coated with the infected cells, air-dried, and fixed with acetone. In the immunofluorescence assays, epitope-specific MAb and fluorescein isothiocyanate (FITC)-conjugated goat anti-mouse IgG (Jackson IR) were used as the primary and secondary antibodies, respectively. The samples were analyzed by using a Leica microscope, and the images were acquired with a Leica digital camera.

## Competing interests

The authors declare that they have no competing interests.

## Authors' contributions

RHH designed the experiment. RHH and NSC carried out most of the experiments and RHH wrote the manuscript. CFQ and YQD prepared rabbit sera against WNV and DV. JYG, XJW, ZJQ, WYC, ZYW, WXL and SH participated part of expremients. ZGB revised the manuscript. All authors read and approved the final manuscript.
